# Screening of Microbial Fermentation Products for Anti-*M. tuberculosis* Activity

**DOI:** 10.3390/ani12151947

**Published:** 2022-07-31

**Authors:** Aikebaier Reheman, Di Lu, Yifan Wang, Xi Chen, Gang Cao, Chuanxing Wan

**Affiliations:** 1 College of Animal Science and Technology, Tarim University, Alar 843300, China; akpar0902@163.com; 2 Bio-Medical Center, Huazhong Agricultural University, Wuhan 430070, China; gcao@mail.hzau.edu.cn; 3 State Key Laboratory Breeding Base for The Protection and Utilization of Biological Resources in Tarim Basin, College of Life Science and Technology, Tarim University, Alar 843300, China; dilu2022@163.com; 4 State Key Laboratory of Agricultural Microbiology, College of Veterinary Medicine, Huazhong Agricultural University, Wuhan 430070, China; giggleral@163.com (Y.W.); chenxi419@mail.hzau.edu.cn (X.C.)

**Keywords:** *M. tuberculosis*, anti-*M.tb*, tuberculosis activity, fermentation products, soil microbial

## Abstract

**Simple Summary:**

*M. tuberculosis (M.tb)* is the main pathogen of tuberculosis (TB). The emergence of multidrug-resistant (MDR) and extensively drug-resistant (XDR) *M.tb* has brought new challenges to the treatment of TB. Therefore, finding new materials for the development of natural anti-TB drugs is crucial to the prevention and treatment of TB. In order to discover new anti-TB drug materials, we isolated microorganisms from the soil and tested the anti-*M.tb* activity of their fermentation products. The results showed that the four fermentation products had anti-*M.tb* activities in vitro and in intracellular bacteria. The qPCR results showed that the four fermentation products down-regulated some growth-essential gene expression of *M.tb.* Thus, we speculated that the fermentation product may exert its anti-*M.tb* effect by down-regulating the expression of the essential genes of *M.tb*.

**Abstract:**

Tuberculosis (TB), caused by *M. tuberculosis (M.tb)*, is the leading infectious cause of mortality worldwide. The emergence of drug-resistant *M.tb* has made the control of TB more difficult. In our study, we investigated the ability of microorganism fermentation products from the soil to inhibit *M.tb*. We successfully identified four fermentation products (*Micromonospora chokoriensis*, *Micromonospora purpureochromogenes*, *Micromonospora profundi*, *Streptomyces flavofungini*) that inhibited the growth of *M.tb* in vitro and in intracellular bacteria at 25 μg/mL MIC. Importantly, the fermentation products decreased some essential gene expression levels for *M.tb* growth. Our data provide the possibility that microbial fermentation products have potential development value for anti-*M.tb* drugs.

## 1. Introduction

Tuberculosis (TB) is a major public health problem worldwide with high morbidity and mortality [1], and it is one of the top 10 causes of human death. TB is an infectious disease caused by *M. tuberculosis (M.tb)*, and approximately one-third of all people worldwide are infected with *M.tb*. The current treatment for active TB is six months of quadruple therapy with four antibiotics, such as rifampicin, isoniazid, ethambutol, and pyrazinamide [2]. The long duration of treatment is partly due to the difficulty in attaining a proper therapeutic drug concentration because of the low barrier across the *M.tb* cell wall. In the past years, resistance to this therapy has risen, and the emergence of multidrug-resistant (MDR) *M.tb* and extensively drug-resistant (XDR) *M.tb* makes the control of TB more challenging [3,4,5,6]. Until today, XDR-TB has no guidelines or evidence that may guide its treatment. To effectively combat these drug-resistant cases, new TB drugs with novel models of action are desperately needed.

Traditionally, natural products (NPs) provide rich sources for the discovery of new antimicrobials [7]. Since the discovery of penicillin [8], antimicrobials have been isolated from NPs or natural product derivatives, such as plants and marine or soil-dwelling microorganisms, among many others. To date, approximately 70% of all available antimicrobials have been derived from nature. In recent studies, NPs and some of their derivatives have shown promising inhibitory activity against *M.tb*, highlighting their potential as a powerful source of new anti-TB drugs. Thus, screening of NPs and their derivatives offers a new approach to the development of novel anti-TB drugs [9,10,11,12,13]. The genus *Micromonospora* has been identified as one of the potential sources of bioactive antimicrobial metabolites with different chemical structures, such as gentamicin, sagamycin, mycinamicin, halomicin, mutamicin, and ivermectin [14]. Some studies reported that *Micromonospora auratinigra* has antibacterial activity [15]. In addition, a *Streptomyces* strain was found in desert sand, which significantly inhibited the growth of *Cryptococcus neoformans* and *Trichophyton mentagrophytes*. In deep fermentation in the laboratory, bacteria produce an antibacterial and antifungal that has been named *Streptomyces flavofungini* [16]. However, it has not been reported whether *Micromonospora* such as *Chokoriensis*, *Purpureochromogenes*, *Profundi*, and *Streptomyces flavofungini* have anti-*M.tb* activity. The antibacterial drugs exert their antibacterial effects by affecting cell wall biosynthesis, protein synthesis, nucleic acid synthesis, energy production, and other emerging targets. The antimicrobials inhibit *M.tb*’s fatty acid biosynthesis by targeting specific genes, such as isoniazid-targeted *KatG*, pyrazinamide-targeted *PncA*, and thionamide-targeted *CmaA2*; rifampicin targets nucleic acid synthesis-related gene *RpoB*; streptomycin and linezolid target the protein synthesis-related genes *RpsL* and *RplC*, respectively [17]. Biotin serves as an essential cofactor of enzymes involved in cellular metabolism, influencing cell growth [18]. Originally isolated from *Streptomyces* sp., biotin targets the biotin-biosynthetic pathway and competitively inhibits the *M.tb*–biotin synthase (*BioB*) [19]. Cyclic heptapeptide isolated from *Streptomyces* marine exhibits bactericidal activity against *M.tb* under salt culture conditions by targeting *ClpC1* [20]. These findings suggest that antibacterial drugs or natural products exert their bacteriostatic effects by affecting the expression of the essential genes of *M.tb*.

In this study, we isolated microorganisms from soil species and fermented them to isolate their fermentation products. We then screened the fermentation products with anti-*M.tb* activity and preliminarily analyzed their antibacterial mechanisms. Among them, four fermentation products had certain anti-*M.tb* activity in vitro through down-regulation of some essential growth genes of *M.tb*.

## 2. Materials and Methods

### 2.1. Medium

We used an ISP 2 liquid medium containing 0.4% yeast extract, 1% wort, 0.4% glucose, and 0.1% NaCI, which was made up to 1 L with distilled water, pH 7.0. We also used an ISP 2 solid medium containing 0.4% yeast extract, 1% wort, 0.4% glucose, 0.1% NaCI, and 1.6% agar, which was made up to 1 L with distilled water, pH 7.0. A seed liquid medium was used that contained 1% starch, 0.3% TSB, 0.4% glucose, 0.4% yeast extract, 1% 1% malt infusion powder, 1% trace elements, and 1.7% agar, made up to 1 L with distilled water, pH 7.0. A fermentation broth medium was used that contained 1% millet, 10% bean sprouts, 1% glucose, 0.5% peptone, 0.25% NaCl, and 0.1% (NH_4_)2SO_4_, made up to 1 L with distilled water, pH 7.0.

### 2.2. Isolation and Identification of Soil Microorganisms

Microorganisms were isolated from the desert alkaline soil in southern Xinjiang, China. After the soil samples were air-dried, they were placed in an electric-heating constant-temperature blast-drying oven (Electric Heating Constant Temperature Blast Drying Oven DHG-9240A Shanghai Qixin Scientific Instrument Co., Ltd., Shanghai. China) for 1 h at 120 °C, and 1 g soil samples were placed in 10 mL of sterile saline and shaken at 156 r/min for 30 min. We added 1 mL of the treated soil sample suspension to a 15 mL tube containing 9 mL of saline and then diluted it into a 10^−2^, 10^−3^ soil suspension. The 100 μL soil suspension was plated on the ISP 2 solid medium and incubated in a 30 °C incubator (Electric Heating Constant Temperature Incubator DHP-9272 Shanghai Qixin Scientific Instruments) for 15 days. After the colonies were visible to the naked eye on the plate, we picked a single colony in the ISP2 liquid medium and incubated it in a 30 °C incubator for 7 days. The total DNA was isolated using the SDS–CTAB method, and 16S rDNA was amplified for sequencing. The sequence results were compared with the EzBioCloud during September 2021. (https://www.ezbiocloud.net, accessed on 13 September 2021) website for species identification. The primers are shown in Table 1.

### 2.3. Extraction of Fermentation Products

For fermentation, the strains were plated on the ISP 2 solid medium and incubated in a 30 °C incubator for 7 days. The colony was picked out and cultured in a seed culture medium at 30 °C for 5 days. We inoculated 4% of the bacterial solution into the fermentation medium and culture at 30 °C for 7 days. We then centrifuged the bacterial solution at 4000 rpm/min for 10 min, and the fermentation products were separated into the fermentation broth and the colony. The fermentation broth was adsorbed with D101 macroporous resin (Beijing Sorabio Technology Co., Ltd., Beijing, China) and washed with 4 column volumes of distilled water three times, 5 min each time, to remove the sugars, salts, and extracellular proteins. We then eluted the broth with 4 column volumes of 30% methanol for 5 min. Finally, 4 column volumes of 95% methanol was used to elute it until colorless, and the eluent was concentrated under reduced pressure by a rotary evaporator to obtain the crude extract of the fermentation products. The crude extract products were dissolved with the final concentration of 0.1% DMSO.

### 2.4. Bacterial Strain and Cell Cultures

For the bacterial strain and cell culture methods, we referred to Chen et al. [21]. The *M.tb* H37Ra was cultured in Middlebrook 7H9 broth (Becton, Dickinson, Franklin Lakes, NJ, USA) and supplemented with 0.5% glycerol, 0.05% Tween 80, and oleic acid albumin dextrose catalase (OADC, Becton, Dickinson, BD, USA). Human monocytes (THP1, ATCCTIB-202) were maintained in an RPMI 1640 medium supplemented with 10% FBS and were differentiated for 24 h using a culture medium containing 40 ng/mL phorbol 12-myristate 13-acetate (PMA) before infection.

### 2.5. Microplate Alamar Blue Assay

The *M.tb* H37Ra was kept at a cultured logarithmic growth stage, OD600 = 0.6–0.7, and 100 μL of bacterial suspension (5 × 10^4^ CFUs) were added to the 96-well cell culture plates. We then added 100 μL of the fermentation products solution to each well. In the positive control group, *M.tb* H37Ra was cultured without the drugs, rifampicin (RIF) was used as the drug control, and DMSO was used as the negative control. After 6 days of culture, 10 μL Alamar Blue (YEASEN Biotech Co., Ltd., Shanghai, China) was added to each well, and incubation was continued for 24 h. After 7 days, the A600 values of the bacterial solutions were measured by a microplate reader (PerkinElmer Enterprise Management (Shanghai) Co., Ltd.,Shanghai, China). To assess the effects of the fermentation on the growth of *M.tb* H37Ra, the A600 value of the fermented products treatment group was compared with the positive control.

### 2.6. Determination of the Minimum Inhibitory Concentration of Selected Fermentation Products

To determine the MICs of the fermented products against *M.tb* H37Ra, 100 μL of bacterial suspension (5 × 10^4^ CFUs) was added to the 96-well cell culture plates. We also added 100 μL of two-fold serial dilutions of the selected fermentation products to each well (0.78–25 μg/mL). The bacterial growth was assessed visually after being cultured for 1 week.

### 2.7. Cell Viability Analysis

The cell viability analysis was measured by a CellTiter 96 AQueous One Solution Cell Proliferation Assay (MTS, Promega, Cat# G3580, Madison, WI, USA) according to the manufacturer’s recommendations. The THP-1 cells were seeded in 96-well plates (3 × 10^4^ cells/100 μL/well) and were differentiated for 24 h with PMA. The cells were then cultured in the presence of various concentrations of selected fermentation products (0, 12.5, 25, and 50 μg/mL) for 48 h, and it was also applied to the 50% effective cytotoxic concentration value determination (CC_50_). After 48 h, 10 μL of the MST kit reagent solution was added to each well, and DMSO was used as a negative control. The plates were incubated for 4 h, and the A490 values were measured using a microplate reader (PerkinElmer Enterprise Management (Shanghai) Co., Ltd.). All cell experiments were conducted using no more than four generations of subculture after thawing the cell stocks. A mycoplasma-free test was performed by using the MycoBlue Mycoplasma Detector (D101-02, Vazyme, Nanjing, China).

### 2.8. Drug Susceptibility Testing against Intracellular M.tb

For the intracellular *M.tb* drug susceptibility test, we referred to Zhou et al. [22]. Briefly, for in vitro antibacterial activity assays, 2 × 10^5^ THP-1 cells were seeded into 24-well plates and were differentiated for 24 h with PMA. The cells were then infected with *M.tb* H37Ra (MOI = 10) for 4 h at 37 °C under 5% CO2. The cells were washed three times with PBS to remove extracellular *M.tb* and supplied with fresh medium with 10% FBS containing selected fermentation products (25 μg/mL). Kanamycin (50 μg/mL) and amikacin (50 μg/mL) were used as positive controls against extracellular *M.tb*. The cells were washed three times with PBS and lysed with sterile 0.1% Tween 80 48 h post-infection. The cell lysates were diluted 10-fold with 7H9 and plated onto 7H11 (Becton, Dickinson) to determine the number of viable bacteria.

### 2.9. Isolated Total RNA and qRT-PCR

We cultured *M.tb* H37Ra in 10 mL at 37 °C and centrifuged (Germany Hettich UNIVERSAL 320 (R) Westphalia, Germany) the bacteria at 4000 rpm/min for 5 min until OD600 reached 0.6–0.7; and then it was washed three times with PBS. The bacteria were suspended with 5 mL fresh 7H9, and we cultured 0.1 mL of these bacteria in 10 mL of fresh 7H9 containing selected fermentation products (25 μg/mL) for 7 days. The total RNAs were extracted from *M.tb* using a RNAiso Plus reagent (Code No. 9109, TaKaRa, Kusats, Japan), and then 1000 ng of the total RNAs were reverse-transcribed to cDNA using ReverTra Ace qPCR RT Master Mix with a gDNA Removal Kit (Vazyme Biotech Co. Ltd., Nanjing, China) according to the manufacturer’s instruction. A SYBR Select Master Mix (TOYOBO, Osaka, Japan) was used for PCR. The PCRs were run on the QuanStudio 6 real-time PCR system, and the relative expression was calculated using the 2-ΔΔCt method. gyrB was used as a control. The primers are shown in Table 1.

### 2.10. Statistical Analysis

The data were analyzed with SPSS software and GraphPad 6.0 software. The differences considered to be statistically significant for *p*-values <0.05, <0.01, <0.001, and <0.0001 are indicated as *, **, ***, and ****, respectively. The statistical significance was determined by a Student’s *t*-test, and each experiment was repeated three times.

## 3. Results

### 3.1. Total of 53 Fermentation Products Were Isolated from the Chickpea Root Nodules and Kaline Soil

**Figure 1:**
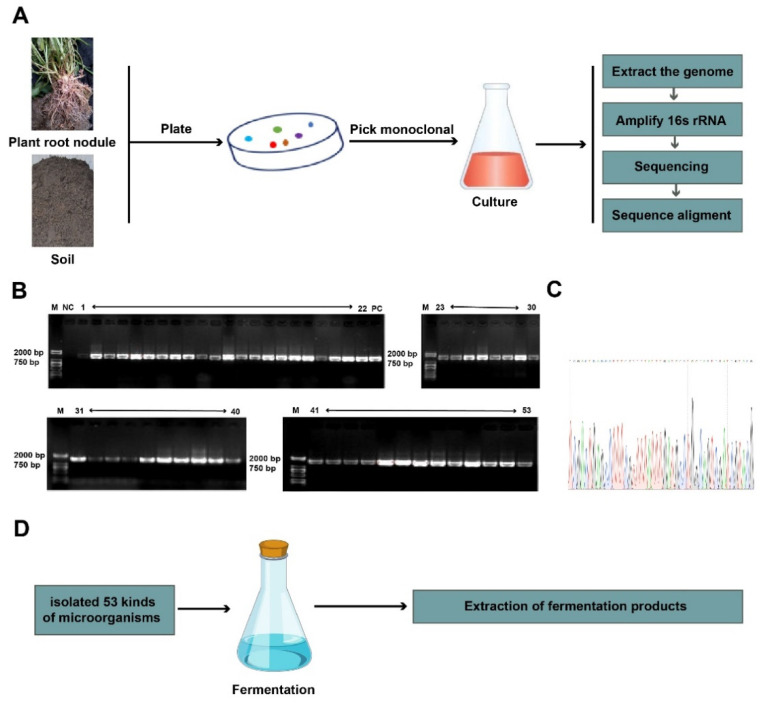
Isolation and identification of 53 microbial fermentation products. (**A**) Schematic of isolation and identification of a plant root nodule and soil microbes. (**B**,**C**) The PCR identification and sequencing of isolated microorganisms. (**D**) Schematic extraction of fermentation products (products 1, 2, 3, 4, 5, 6, 7, 8, 9, 10, 11, 12, 13, 14, 15, 16, 17, 18, 19, 20, 21, 22, 23, 24, 25, 26, 27, 28, 29, 30, 31, 32, 33, 34, 35, 36, 37, 38, 39, 40, 41, 42, 43, 44, 45, 46, 47, 48, 49, 50, 51, 52, and 53) from natural microorganisms.

In this study, we isolated soil and nodule microorganisms by using traditional culture methods. We then identified the species of microorganisms by using a single colony culture, DNA extraction, PCR amplification, and sequencing (Figure 1A–C). We found a total of 53 species of microorganisms through species identification (Table 2), and we obtained 53 fermentation products from 53 strains (Figure 1D).

### 3.2. Screening the Fermented Products against M.tb In Vitro

**Figure 2:**
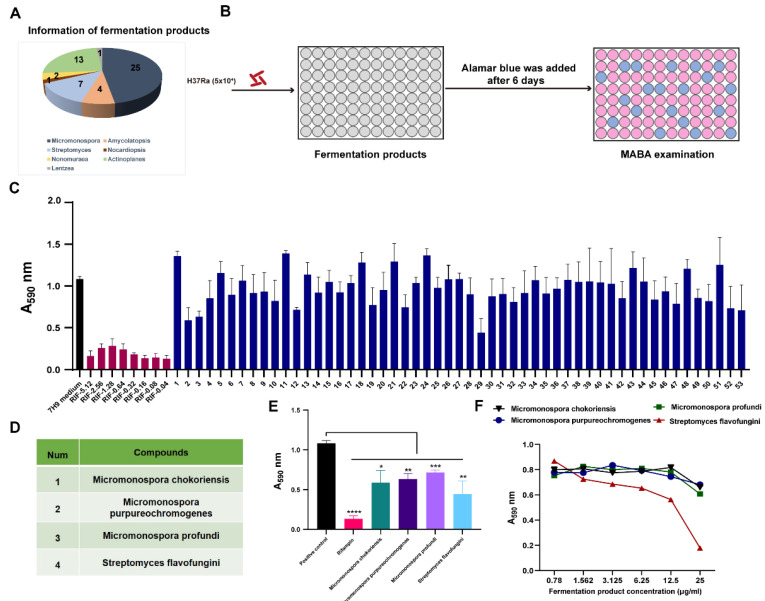
In vitro screening of fermentation products against *M.tb* H37Ra. (**A**) The distribution of each fermentation product in the natural microorganisms. (**B**) Schematic of screening each fermented product inoculated with a suspension of *M.tb* H37Ra in 96-well microplates for 7 days, with antibacterial effects determined using an Alamar Blue assay. (**C**) The anti-*M.tb* H37Ra effects of each fermented product. (**D**,**E**) Four fermentation products were selected with anti-*M.tb* activity, and rifampicin (RIF) was used as a positive control. (**F**) The determined MIC of selected fermented products. Data presented as mean ± SEM. All experiments were performed in triplicate. The values of *, *p* < 0.05; **, *p* < 0.01; ***, *p* < 0.001; and ****, *p* < 0.0001 were considered statistically significant differences.

In our study, the *M.tb* H37Ra was used to screen new anti-TB fermentation products. Our analysis results showed that the fermentation products were from seven microorganisms, namely, *Micromonospora*, *Streptomyces*, *Nonomuraea*, *Lentzea*, *Amycolatopsis*, *Nocardiopsis*, and *Actinoplanes* (Figure 2A). The *M.tb* H37Ra was cultured in 96-well cell culture plates containing 25 μg/mL of each fermentation product, and the antibacterial activity was measured using Alamar blue assays (Figure 2B). The screening of the fermentation products showed that 4 fermentation products out of the 53 candidates inhibited *M.tb* growth in vitro (Figure 2C). These fermentation products include *Micromonospora chokoriensis*, *Micromonospora purpureochromogenes*, *Micromonospora profundi*, and *Streptomyces flavofungini* (Figure 2D). The significance analysis showed that there were significant differences between the anti-*M.tb* activity of the selected fermentation products and the anti-*M.tb* activity of the positive control (Figure 2E). The MICs’ results showed that the selected fermentation products showed activity against *M.tb* with MICs of 25 μg/mL. These data indicated that the fermentation products could have potential anti-*M.tb* activity.

### 3.3. Intracellular Bactericidal Effects of Fermentation Products

**Figure 3:**
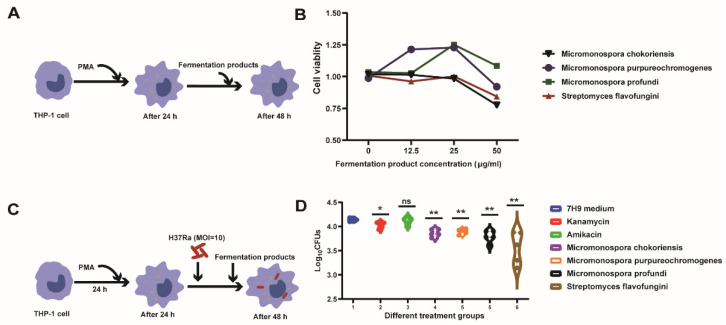
Cytotoxicity detection and intracellular anti-*M. tb* activities of fermented production. (**A**,**B**) A cell viability assay was used to detect the toxicity of selected fermented products on THP-1 cells. (**C**,**D**) Procedure for testing the susceptibility of intracellular fermented products in THP-1 cells and the CFU enumeration of *M. tb* H37Ra in THP-1 cells treated with individual fermented products for 48 h post-infection. Data presented as mean ± SEM. All experiments were performed in triplicate. The values of *, *p* < 0.05; **, *p* < 0.01 were considered statistically significant differences.

The cytotoxicity of the selected fermentation products (*Micromonospora chokoriensis*, *Micromonospora purpureochromogenes*, *Micromonospora profundi*, and *Streptomyces flavofungini*) was examined by using the MTS method. A decrease in A_490_ indicated cytotoxicity toward in the THP-1 cell. For the cell viability assays, the THP-1 cells were differentiated with PMA, and the selected fermentation products were added to each well at various concentrations (Figure 3A). The results showed that the selected fermentation products were not cytotoxic to the THP-1 cells at concentrations of 12.5 μg/mL and 25 μg/mL (Figure 3A). Considering the MIC concentration in vitro, we chose 25 μg/mL to detect the anti-intracellular *M.tb* activity. Next, we examined the activities of the selected fermentation products on the clearance of the intracellular *M.tb* in the THP-1 cells (Figure 3C). The results showed that the number of CFUs from the fermentation products’ treatment cells was significantly lower than those of the 7H9 control (Figure 3D). These results indicated that the selected fermentation products could clear intracellular *M.tb* at a MICs concentration (25 μg/mL).

### 3.4. Effect of Fermentation Products on M.tb’s Gene Expression

**Figure 4:**
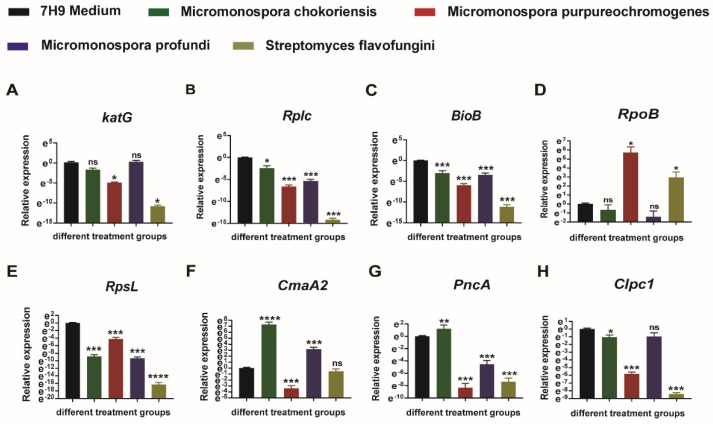
The expression level of *M.tb* essential genes after treatment with four selected fermentation products. Data presented as mean ± SEM. All experiments were performed in triplicate. The values of *, *p* < 0.05; **, *p* < 0.01; ***, *p* < 0.001; and ****, *p* < 0.0001 were considered statistically significant differences.

To better understand the cause of the four fermentation products’ (*Micromonospora chokoriensis*, *Micromonospora purpureochromogenes*, *Micromonospora profundi*, and *Streptomyces flavofungini*) resistance to *M.tb*, we examined the mRNA expression levels of essential genes for *M.tb* growth after being treated with fermentation products. Biological processes, such as protein synthesis, fatty acid biosynthesis, nucleic acid synthesis, and energy production, are critical to the growth of *M.tb*, and most antibiotics also affect the growth of *M.tb* through these pathways. Thus, we selected genes (*katG*, *Rplc*, *BioB*, *RpoB*, *RpsL*, *CmaA2*, *PncA*, and *Clpc1*) that were involved in those biological processes. Our results showed that the mRNA expression level of *Rplc*, *BioB*, *RpsL*, and *Clpc1* was significantly decreased, compared with the control group, after being treated with *Micromonospora chokoriensis* (Figure 4B,C,E,H). The mRNA expression level of the *katG*, *Rplc*, *BioB*, *RpsL*, *CamA2*, *PncA*, and *Clpc1* genes was significantly decreased, compared with the control group, after being treated with *Micromonospora purpureochromogenes* (Figure 4A–H). The expressions of *Rplc*, *BioB*, *RpsL*, and *PncA* were down-regulated by *Micromonospora profundi* (Figure 4B,C,E,G). The expressions of *katG*, *Rplc*, *BioB*, *RpoB*, *RpsL*, *PncA*, and *Clpc1* in the *Streptomyces flavofungini* treatment group were significantly lower than those in the control group (Figure 4A–E,G,H). However, there were also some genes whose expression was not affected by the fermentation products. At the same time, we also observed that several genes were up-regulated in some cases; for instance, *Micromonospora chokoriensis* up-regulated the expression of the genes *CmaA2* and *PncA* (Figure 4F,G), and *Micromonospora purpureochromogenes* and *Streptomyces flavofungini* up-regulated the expression of the gene *RpoB* (Figure 4D). However, a small number of genes were up-regulated, and most genes were still effectively suppressed by the four fermentation products.

## 4. Discussion

As a zoonotic infectious disease, tuberculosis has seriously affected human health and the sustainable development of animal husbandry. In recent years, *M.tb*’s antibiotic resistance is becoming increasingly common, and disease control is challenging [23]. Early studies reported that natural products are important sources for anti-TB drug discovery and have provided several novel molecular scaffolds. Most of these active compounds were identified through phenotype screening against different mycobacteria strains [24,25]. Several natural products have advanced to market, including streptomycin, cycloserine, kanamycin, rifampicin, capreomycin, and amikacin. Therefore, screening secondary metabolites produced by plants and soil microorganisms provides the most possibility for the discovery of anti-TB drugs. In our study, we isolated 53 fermentation products from soil microorganisms, with half of them being members of *Micromonospora*. Recent studies have reported that *Micromonospora auratinigra* has bacteriostatic effect on Staphylococcus aureus, Bacillus subtilis, Proteus vulgaris, Escherichia coli, Pseudomonas aeruginosa, and Mycobacterium abscessus [15] and that *Streptomyces euryhalinus* shows antibacterial activity [26]. Therefore, we examined the antibacterial activity of 53 microbial fermentation products. Our results showed that 4 of the 53 fermentation products (*Micromonospora chokoriensis*, *Micromonospora purpureochromogenes*, *Micromonospora profundi*, and *Streptomyces flavofungini*) were able to inhibit the growth of *M.tb* in vitro and intracellularly and were non-toxic to cells at the MIC level.

Next, we further analyzed the mechanism of action of these four fermentation products that inhibited the growth of *M.tb*. Previously, some reports explained that the natural products inhibited *M.tb*’s growth by targeting essential genes, which are related to cell wall synthase, protein production, energy generation, and nucleic acid synthesis [27]. To better understand how these four fermentation products affect the growth of *M.tb* in vitro and intracellularly, we further examined the expression of some genes (*katG*, *Rplc*, *BioB*, *RpsL*, *RpoB*, *CmaA2*, *PncA*, and *Clpc1*) after being treated with four fermentation products; these genes were essential genes for the growth of H37Rv [28,29,30]. Our results showed that *Micromonospora chokoriensis* could inhibit the gene expression of *Rplc*, *BioB*, *RpsL*, and *Clpc1* in vitro. Treatment with *Micromonospora purpureochromogenes* inhibited the expression of *KatG*, *Rplc*, *BioB*, *RpsL*, *CmaA2*, *PncA,* and *ClpC1*. The *Rplc*, *BioB*, *RpsL*, and *PncA* expressions were inhibited after being treated with *Micromonospora profundi*, and *katG*, *Rplc*, *BioB*, *RpsL*, *PncA*, and *Clpc1* expressions were down-regulated by *Streptomyces flavofungini*. *Rplc* is a ribosomal L3 protein involved in protein synthesis in *M.tb*, and it was identified as a novel target in the linezolid (LZD)-resistant *M.tb* strains [31]. Biotin is an important cofactor for enzymes involved in cellular metabolism and for enzymes affecting cell growth. *BioB* is a biotin synthetase gene, and some studies reported that acidomycin could play an anti-*M.tb* role by targeting *BioB* [19]. The *RpsL* encoding for ribosomal subunit 16S rRNA was involved in protein synthesis in *M.tb* and was associated with resistance to streptomycin [32]. Rv3596c (*ClpC1*) suggests that intrinsic ATPase activity is important for protein homeostasis, and the *ClpC1* gene is thought to be required for *M.tb* cell growth [33]. It is reported that *KatG*-mediated oxidation could reduce the sensitivity of bacteria to Kanamycin and mainly involves fatty acid biosynthesis [34]. Fatty acid oxidation is an important carbon source for *M.tb*. *CmaA2* is a mycolic acid synthase, and mycolic acids make the cell wall less permeable, contribute to antibiotic resistance and host immunomodulation, and protect from injury [35]. *PncA* also involves fatty acid biosynthesis and encodes pyrazinamidase, an enzyme which converts the drug pyrazinamide to the active form pyrazinoic acid [36]. The above genes are all involved in the important life process of *M.tb*. Therefore, we suggested that *Micromonospora chokoriensis* may affect the transcription of the above genes to exert its antibacterial effects. It turned out that the four fermentation products repressed roughly the same genes, but different numbers of genes were repressed, and it even up-regulated the expression of some genes. For instance, CmaA2, PncA, and RpoB were up-regulated by *Micromonospora chokoriensis* and *Micromonospora purpureochromogenes.* Our results showed that the four fermentation products had different antibacterial effects, with *Micromonospora purpureochromogenes* and *Streptomyces flavofungini* being the most effective. This finding is consistent with the gene expression results, which showed that *Micromonospora purpureochromogenes* and *Streptomyces flavofungini* suppressed the largest number of genes. The bacteriostatic effect may be due to differences in the degree and number of genes inhibited by the fermentation products. Thus, we suggest that selected fermentation products may inhibit the growth of *M.tb* by affecting the expression of some essential genes.

## 5. Conclusions

In conclusion, we isolated and identified some microorganisms from soil, fermented them, and extracted their fermentation products. The anti-*M.tb* effect of the fermentation products was detected, and four fermentation products were found to have anti-*M.tb* activity. Further analysis found that these four fermentation products exerted their anti-*M.tb* effects by inhibiting the expression of genes essential for *M.tb* growth. Our data provide new possibilities for the development of new anti-TB drugs and elucidate their mechanism of action, which could help combat MDR and XDR-TB.

## Figures and Tables

**Table 1:** qPCR primer sequences.

Gene	Forward Primer	Reverse Primer
clpC1	5′-GTCGTCCTGGCTCAGGAAGA-3′	5′-GACCTGACTGCGCACACCTT-3′
katG	5′-TACAGAAACCACCACCGGAG-3′	5′-TAGTCGAACGCCGCACCCAT-3′
pncA	5′-GACGTGCAGAACGACTTCTG-3′	5′-ATAGTCCGGTGTGCCGGAGA-3′
cmaA2	5′-ACAAGCGGCACGCAGCTCAA-3′	5′-TACTGCGCCTCTTCCAGCGT-3′
rpoB	5′-ACAGCCGCTAGTCCTAGTCC-3′	5′-CGAACCGATCAGCCACTCGA-3′
rpsL	5′-TCGGGACAAGATCAGTAAGG-3′	5′-ATGTACGCCGTGACCTCGAC-3′
rplC	5′-TATGACGCAGGTATTCGACG-3′	5′-GACCTTGCGTGGGCTGATCT-3′
bioB	5′-ATGGCGGGAACAACTCGGAC-3′	5′-TGATGATGCCTTCGACCTCG-3′
16S rDNA	5′-AGAGTTTGATCCTGGCTC-3′	5′-CGGCTACCTTGTTACGACTT-3′

**Table 2:** All isolated and identified microorganisms.

Num.	Microorganisms	Num.	Microorganisms
1	*Micromonospora taraxaci*	28	*Nonomuraea kuesteri*
2	*Micromonospora chokoriensis*	29	*Streptomyces flavofungini*
3	*Micromonospora purpureochromogenes*	30	*Streptomyces griseoincarnatus*
4	*Micromonospora halophytica*	31	*Actinoplanes alaerensis*
5	*Micromonospora lutea*	32	*Streptomyces mangrovi*
6	*Micromonospora saelicesensis*	33	*Streptomyces griseus* subsp. *Griseus*
7	*Micromonospora inaquosa*	34	*Amycolatopsis roodepoortensis*
8	*Micromonospora vinacae*	35	*Streptomyces flavofungini*
9	*Micromonospora zamorensis*	36	*Lentzea jiangxiensis*
10	*Micromonospora chalea*	37	*Amycolatopsis lurida*
11	*Micromonospora terminaliae*	38	*Amycolatopsis lurida-1*
12	*Micromonospora profundi*	39	*Nonomuraea harbinensis*
13	*Micromonospora spongocola*	40	*Micromonospora sediminimaris*
14	*Micromonospora auratinigra*	41	*Actinoplanes alaerensis*
15	*Micromonospora cremea*	42	*Micromonospora andamanensis*
16	*Micromonospora gifhorensis*	43	*Actinoplanes*
17	*Micromonospora phytophia*	44	*Actinoplanes xinjiangensis*
18	*Micromonospora andamanensis*	45	*Actinoplanes utahensis*
19	*Micromonospora fiedleri*	46	*Actinoplanes brasiliensis*
20	*Micromonospora soli*	47	*Actinoplanes rectilineatus*
21	*Micromonospora oryzae*	48	*Actinoplanes cyaneus*
22	*Micromonospora dersiti*	49	*Actinoplanes atraurantiacus*
23	*Micromonospora inositola*	50	*Actinoplanes lutulentus*
24	*Amycolatopsis marina*	51	*Actinoplanes abujensis*
25	*Streptomyces formicae*	52	*Actinoplanes missouriensis*
26	*Nocardiopsis terrae*	53	*Actinoplanes abujensis*
27	*Streptomyces griseorubens*		

## Data Availability

The data supporting the reported results are contained within the article.
